# Victim’s profile analysis reveals homicide affinity for minorities and the youth

**DOI:** 10.5249/jivr.v2i2.50

**Published:** 2010-06

**Authors:** Evelio Velis, Graham Shaw, Alan S. Whiteman

**Affiliations:** ^*a*^College of Health Sciences, Barry University, USA.; ^*b*^College of Health Sciences and School of Podiatry Medicine, Barry University, USA.; ^*c*^College of Health Sciences, Barry University, USA.

## Abstract

**Background:**

In this study we have examined the risk of death by homicide in Miami-Dade County and Broward County (BC); and examined the association between socioeconomic status and homicide while describing victim's typical characteristics such as age, gender, race/ethnicity, and type of injury.

**Methods:**

Data was collected from the County's Medical Examiner's Offices, Census Bureau, and Federal Bureau of Investigation between 2004 and 2007.

**Results:**

There has been an increase in the risk of dying by homicide in the studied area; the homicide rate for the selected period was two times higher than the national average. Although Black Non-Hispanics count for 19% of the population of Miami-Dade County and 23% in Broward County, 56% and 53% of homicide victims are among this ethnic group in Miami-Dade County and Broward County respectively. Hispanics were more at risk of being a victim of homicide in 2007 than they were in 2004.

**Conclusions:**

A substantial proportion of the homicide victims were 22 years of age or younger. In fact, the homicide victims' average age has been steadily decreasing in recent years. The drastic increase in the risk of death by gunshot among young Black non-Hispanic and Hispanic residents warrants cause for concern.

## Introduction

The word homicide invokes scenes from horror movies and forensic science-related TV shows with white-chalk outlines of bodies; none of these images really portrays homicide as a serious health related problem. Since the lives of victims, families, and friends are affected by this despicable act of violence, this topic should be considered relevant to the public health prevention agenda. Though homicide rates nationwide have remained unchanged over the last several years,^1^ in Miami-Dade County the homicide rate increased by 45% from 2005 to 2006,(according to Miami-Dade County’s Mayor Carlos Alvarez). Despite the increase in homicide rate in Miami-Dade County there has only been an 8% increase in murder arrests over the same period. ^2^ In fact based on a Miami New Times report published in 2004, Miami shows the highest rate of violent crime in America. ^3^

Homicide is highly prevalent in our communities, with the potential to drastically affect anyone at any time. While South Florida, specifically Miami, is known for its beautiful beaches, fashion, glamour, and music, the area has a dark side in stark contrast to the glamorous high-life. Crime, including homicide, is on the increase.

Homicide should not be exclusively the concern of law enforcement agencies, politicians, or elected public officials; it should also be of interest to health professionals and public health authorities. Therefore, the main goal of the current study is to raise the level of awareness within our community of the growing homicide problem. This will allow the entire community to actively participate in significantly reducing the risk of homicides in the area while at the same time providing social justice.

## Methods

**Data**

We conducted a cross-sectional study of homicide victims’ characteristics in Miami-Dade County and Broward County between 2004 and 2007. The data was obtained from the Miami-Dade and Broward counties Medical Examiners’ offices, the U.S. Census Bureau, and the Federal Bureau of Investigation. The variables included in the database were: gender, race/ethnicity, age, type of injury, and selected socioeconomic factors.

Victims were classified in the original database as only Whites or Blacks. Conscious of the importance of Hispanics in the area the data was reclassified in order to include the missing race/ethnicity information. A method was devised using Latin/Hispanic last name recognition in order to adjust for the insufficient information of the original data set; then the victims were reclassified based on race/ethnicity.

The age of the victim was measured in years and the following age groups were constructed for the analysis: “less than 14 years old”, “14 to 17 years old”, “18 to 24 years old”, and “25 years old or more”.

Victims of homicide were also classified according to the type of injury received. Description and analysis were based on the most frequent types of injuries: gunshot, stabbing, and other. The Medical Examiners’ database includes under the “other” category: ‘assaulted’, ‘battered’, ‘beaten’, ‘asphyxia’, ‘victims of arson’, and ‘injured by other(s)’.

The four-year period homicide rates were calculated based on the average of homicide victims and average of the counties’ mid-year population for the 2004 to 2007 period. Annual homicide rates were calculated based on the number of victims and the mid-year population for each year; all rates were crude rates per 100,000 population.

The data related to the selected socioeconomic factors, education, poverty, and unemployment, were extracted from the Census Bureau publications. “Poverty” is defined as the percentage of individuals living under the poverty level based on the Census Bureau criteria.

**Statistical Analysis**

Data was organized and validated using the Statistical Package for Social Sciences (SPSS) 17.0® version. Indicators of central tendency and dispersion [means (M), standard deviations (SD), standard errors of the mean (SEM), and 95% confident intervals] were estimated for quantitative variables while frequencies and percentages were used for qualitative variables. Student’s t tests and Analysis of Variance (ANOVA) were performed to identify differences between groups where appropriate. In order to identify associations between exposure (risk factors) and outcomes (death by homicide) the Chi-Square test was used; Odds Ratios were estimated to establish the risk levels among different groups. A correlation analysis was also performed exploring relationships between homicide and socioeconomic indicators by state. Correlation analyses were performed using education, poverty, and employment related data for all states of the U.S.

## Results

Between 2004 and 2007, the total number of homicide victims in both Miami-Dade County and Broward County combined during the selected four-year period was 1267; an average of 317 deaths per year with a homicide crude rate for both counties around 15 deaths per 100,000 population; 71% of homicides across the two counties of occurred in Miami-Dade County. Homicide victims in Miami-Dade County and Broward County increased by 36% in 2007 when it was compared to 2004. Miami-Dade County had 24% more victims of homicide in 2007 (252) than in 2004 (204). In Broward County, victims of homicide increased by 72% in 2007 (119) when compared to 2004 (69).

The homicide rates for the four-year period combined in our study were 9.45 and 5.13 victims per 100,000 population for Miami-Dade County and Broward County respectively; nearly two times higher in Miami-Dade County, where the risk of dying by homicide is more than twice the rest of the nation.

**Figure1: Homicide Rates, Miami-Dade County and Broward County (Years 2004-2007):**
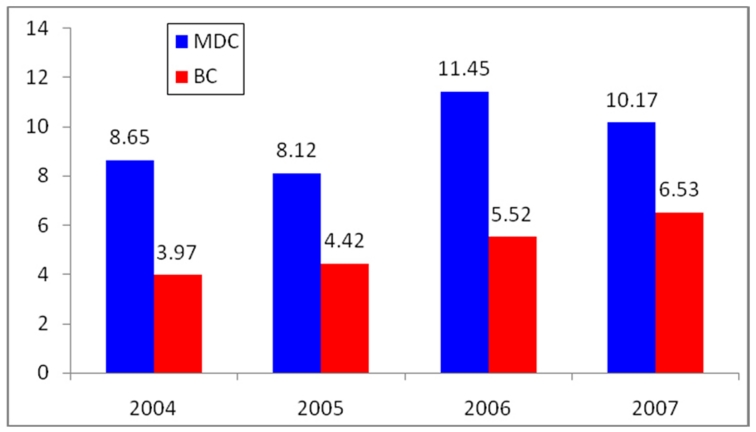
						Note: Rates per 100,000 populations. Rates were calculated using the number of victims and mid-year population for each of the four years period 2004, 2005, 2006, and 2007.

Based on our data the risk of dying by homicide has been increasing in both counties since 2004. In Miami-Dade County the risk of being a homicide victim was 18% higher in 2007 (10.17) than in 2004 (8.65) and in Broward County the risk of dying by homicide has increased by almost 50% over the same time period. The probability of being a victim of homicide in Miami-Dade County, during 2004, 2005, 2006, and 2007 was consistently around two times higher than in Broward County (Figure 1).

**Gender**

Male victims constituted the vast majority of all victims of homicide in both counties; around 80% of all victims were men. Broward County had proportionally more female victims than Miami-Dade County. The proportion of female victims in Broward County (23.8%) was significantly higher than in Miami-Dade County (17.6%), (O.R. = 1.46, χ2 = 6.35, p<0.05). The homicide rate among males increased from 2004 to 2007 in both counties. In Miami-Dade County, the risk of being a male victim escalated by 27% from 2004 to 2007; there was an 80% increase in Broward County during the same period of time. We did not find any significant change in the risk of dying by homicide among females in either of the counties studied.

The risk of dying by homicide in Miami-Dade County in 2004 was four times higher for males. In 2007, the risk of dying by homicide was 6.2 times higher for males than females. In Broward County, the male victims’ relative risk of dying by homicide increased from 2.4 in 2004 to 3.4 in 2007. Not only did males have a higher risk of dying by homicide but this higher risk continues to increase in both counties.

**Race/Ethnicity**

**Figure2: Homicide Victims and Population Distribution by Race/ Ethnicity. Miami-Dade County and Broward County, Four-Year Period 2004-2007.**
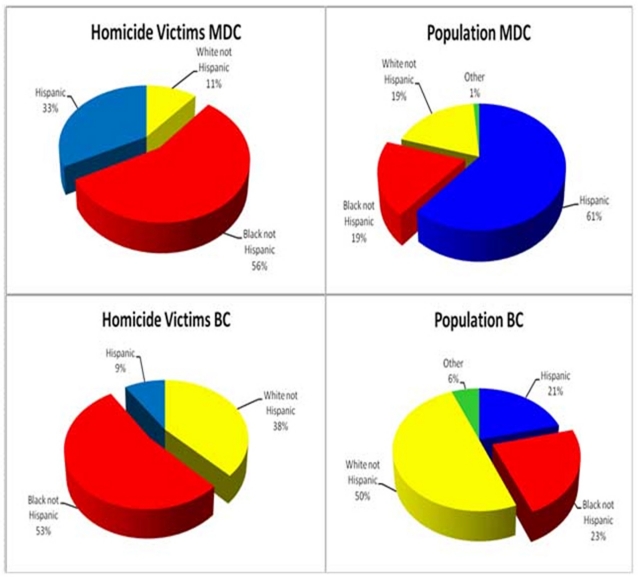


Black Non-Hispanics died by homicide across both counties at a significantly higher proportion than White Non-Hispanics and Hispanics combined. Even though Black Non-Hispanics, according to the Census Bureau, account for only 19% of the Miami-Dade County population, 56% of homicide victims are among this ethnic group. The same proportional disparity is seen in Broward County. In this county, 53% of the homicide victims are Black Non-Hispanics, while only 23% of the Broward County population belongs to this ethnic group (Figure 2).

In Miami-Dade County, during the selected four-year period, females accounted for 25% and 24% of all homicide victims among White Non-Hispanic and Hispanics respectively. The proportion of female victims, in Miami-Dade County was two times higher among White Non-Hispanic (O.R. = 2.27, χ2 = 9.54, p<0.01) and Hispanics (O.R. = 2.20, χ2 = 17.29, p<0.001) than Black Non-Hispanics (12.5%). The proportion of White Non-Hispanic female victims in Broward County was higher (30%) than that of any other female race/ethnic group, (O.R. = 1.64, χ2 =3.96, p<0.05).

During the four-year selected period on average, the homicide rate for Black Non-Hispanics in Miami-Dade County was 28.4 deaths per 100,000 population, significantly higher than Hispanics (5.0) and White Non-Hispanics (6.2) respectively. In Broward County, the risk of dying by homicide among Black Non-Hispanics was 11.6 deaths per 100,000 population and 3.1 greater than Hispanics (2.2) and White Non-Hispanics (3.8) (Table 1).

**Table:** Table 1: **Homicide Rates by Race/Ethnicity Miami-Dade County & Broward County, Four-Year Period 2004-2007**

	Hispanic		Black Non-Hispanics		White Non-Hispanics	
	MDC	BC	MDC	BC	MDC	BC
Average Cases	72	8	126	48	24	34
Rates	5.0	2.2	28.4	11.6	6.2	3.8

Note: Rates per 100,000 populations. Rates were calculated using the average victims and average mid-year population for the four years period 2004, 2005, 2006, and 2007.

**Age**

**Figure 3:**
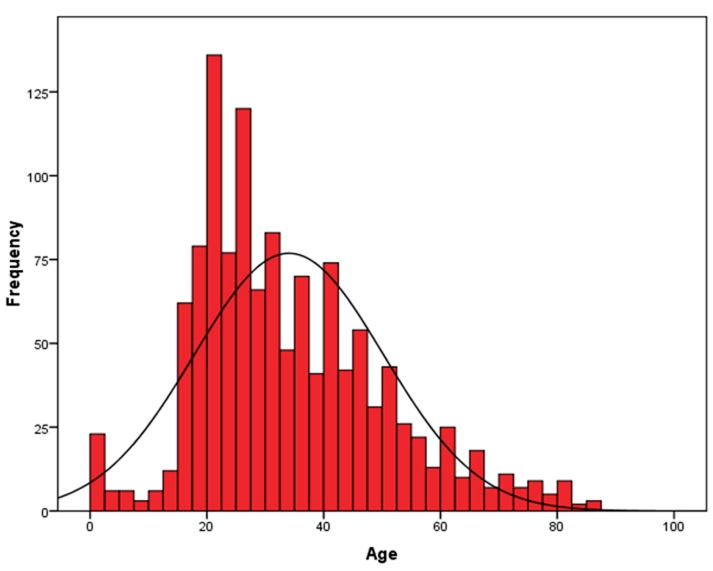
Figure 3: Victims Distribution by Age. Miami-Dade County & Broward County, Four-Year Period, 2004-2007.

The average age of all homicide victims from Miami-Dade County and Broward County between 2004and 2007 was 34 years, with a 95% confidence interval of 33 to 35 years old; 25% of all victims were 22 years old or younger (Figure 3).

In Miami-Dade County, victims of homicide were considerably younger in 2007 (N = 248, M = 34, SEM = 0.95) than they were in 2004 (N = 200, M = 36, SEM =1.14). In Broward County, the opposite trend occurred; victims of homicide were older in 2007 (N = 119, M = 35, SEM = 1.67) than they were in 2004 (N = 69, M = 32, SEM = 2.32).

**Figure 4:**
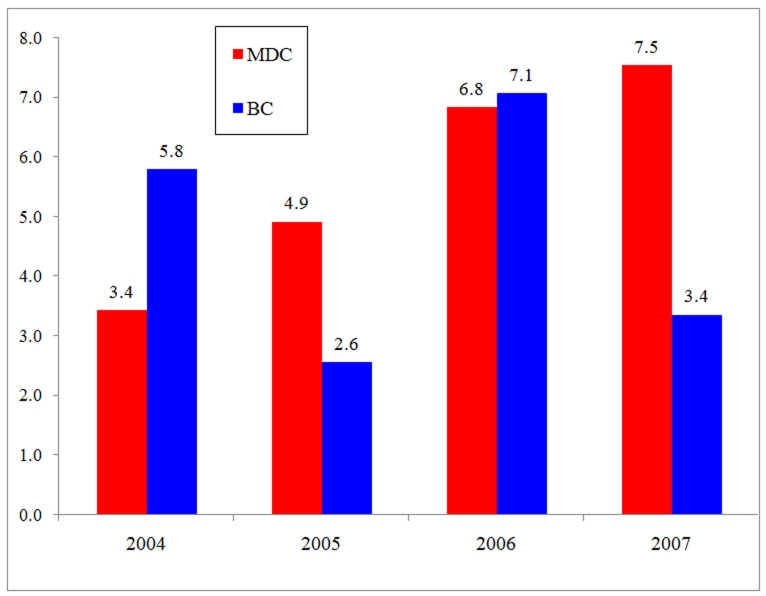
Figure 4: Proportion of Homicide Victims 14 to 17 Years Old Miami-Dade County & Broward County, 2004, 2005, 2006, and 2007.

The proportion of victims, aged 14 and 17 has been rising in Miami-Dade County since 2004. In 2004, the proportion of homicide victims aged between 14 and 17 years old was 3.4% of all homicide victims, though this figure increased to 7.5% in 2007. In Broward County, no clear trend was observed over the same time period. In general, there was an increasing trend of victims among this very young group in both counties from 2004 to 2007 (Figure 4).

The proportion of victims younger than 14 years old was significantly higher in Broward County (6%) than in Miami-Dade County (3%), (O.R. = 2.12, χ2(1) = 6.66, p<0.05). Eleven percent of all homicide victims in Broward County between 2004and 2007 were 17 years old or less; in Miami-Dade County, victims younger than 18 years old made up 9% of total victims. Over 30% of all victims in both Miami-Dade County and Broward County, were younger than 25 years old (Table 2).

**Table:** Table 2: **Homicide Victims by Age Group. Miami-Dade County & Broward County. Four-Year Period 2004-2007.**

Age Groups	MDC		BC		Total	
	Victims	%	Victims	%	Victims	%
Less than 14 years old	26	2.9	22	6.0	48	3.8
14 to 17 years old	53	6.0	17	4.7	70	5.6
18 to 24 years old	215	24.3	77	21.1	292	23.4
25 years old or more	590	66.7	249	68.2	839	67.2
Total	884	100	365	100	1249	100

Proportionally more children died as victims of homicide in Broward County than in Miami-Dade County during the selected four-year period. In Miami-Dade County 1.5% of the victims were children 5 years of age or younger. In Broward County, 4.7% of victims were less than five years old.

**Age/Gender**

Male victims of homicide in Miami-Dade County over the four-year period investigated had a mean age of 33 years (SEM = 0.55) and were significantly younger than female victims (M = 38, SEM = 1.40), t(903) = -3.12, p<0.01. The mean age of both male and female homicide victims in Broward County was 34 years,; 25% of the female victims in Broward County were 19 years old or younger while in Miami-Dade County the 25th percentile was positioned at 25 years old for the same gender. Female victims in Broward County (M=34, SEM=2.47), were considerably younger than those in Miami-Dade County (M=38, SEM=1.40).

**Age/Ethnicity**

Black Non-Hispanic victims of homicide in Miami-Dade County, had mean age of 30 years old, (N=498, SEM=0.58) and were significantly younger than White Non-Hispanic(N=92, M=38, SEM=1.68) and Hispanic (N=289, M=39, SEM=1.00), F (3,880) = 27.05, p <0.01, as shown intable 3.

**Table:** Table 3: **Mean Age by Race/Ethnicity and County, four -Year Period 2004-2007**

Indicator	Race/Ethnicity			
	White Non-Hispanics	Black Non-Hispanics	Hispanic	All Races
MDC	38.0 (1.68)	30.0** (0.58)	39.4 (1.00)	34.0 (0.52)
BC	40.6 (1.80)	29.8** (1.12)	32.3 (1.89)	34.2 (0.94)

Standard errors of the mean (SEM) are in parenthesis. ** p<0.01

In Broward County, the Black Non-Hispanics victims’ average age was 29.8 years old (SEM = 1.12), not significantly different from the Black Non-Hispanic homicide  victims in Miami-Dade County. In Broward County, the Black Non-Hispanic homicide victims were significantly younger than the White Non-Hispanics victims of homicide (N=135, M=40.6, SEM=1.80), F (3,361) = 10.70, p< 0.001 (Table 3) though not significantly younger than Hispanic victims of homicide in this county.

In Miami-Dade County, proportionally, more Black Non-Hispanics were victims of homicide than any other race/ethnic category for all the selected age groups (χ2(2) = 50.2, p<0.001). The highest proportion (76%) is among victims 18 to 24 years of age, significantly higher than the rest of age groups and any other race or ethnicity (O.R. = 3.18, χ2(1) = 44.7, p<0.001).

In Broward County, significantly more Black Non-Hispanic individuals were victims of homicide for all selected age groups (χ2(2) = 15, p<0.05). White Non-Hispanics 25 years of age or older (43%) and younger than 14 years old (36%) were killed relatively more than any other selected age group.

The risk of dying in Miami-Dade County by homicide during the selected four-year period was 10 times higher for Black Non-Hispanics 18 to 24 years of age (64.9 deaths per 100,000 populations) than Hispanics (6.6), and 6 times higher than that for White Non-Hispanics (11.0) of the same age. In Broward County, the homicide victimization rate for Black Non-Hispanics (24 deaths per 100,000 population) was 4.5 and 2.8 times greater than Hispanics (5.3) and White Non-Hispanics (8.5) respectively.

There was an increasing trend in the number of victims of homicide in Miami-Dade County, especially among young Black Non-Hispanics. Homicide victims in this race/ethnic group between the ages of 18 and 24 increased by 58% from 2004 (56.2 deaths per 100,000 population) to 2007 (88.6 deaths per 100,000 population). Among Hispanics of the same age group, the risk of dying by homicide increased over the same time period; the risk was 200% greater in 2007 (10.0 deaths per 100,000 population) than in 2004 (3.3 deaths per 100,000 population). A decrease of 66% was observed among White Non-Hispanics over the same period.

The risk of being killed in Broward County increased among Black Non-Hispanics and Hispanics of 18 to 24 years of age from 2004 to 2007. There were no Hispanic victims reported in 2004; yet in 2007, (7.7 deaths per 100,000) this ethnic group was 2.4 times more likely to be killed than it was in 2005 (3.2 deaths per 100,000). There was a decrease among White Non-Hispanics from 2004 (12.7 deaths per 100,000) to 2007 (7.0 deaths per 100,000).

**Type of Injury**

During the four-year period from 2004 to 2007, Miami-Dade County had a total of 902 deaths due to homicide, 75% of which were victims of gunshot injuries; in Broward County 64% of victims died because of gun-related injuries.

Deaths due to gun-related injuries were significantly more prevalent in Miami-Dade County than in Broward County (χ2 (1) = 15.31, p<0.001). Conversely, stabbing-related deaths were more prevalent in Broward County (12%) than in Miami-Dade County (9%) and lower than the nation for the same period of time, (χ2(1) = 3.53, p>0.05).

Blacks accounted for 63%, Whites 9%, and Hispanics 28% of deaths by gunshot in Miami-Dade County. Broward County data revealed that Blacks accounted for 61%, Hispanics 9%, and Whites 30% of homicides by gunshot. For both counties, Blacks have a higher incidental rate of death by gunshot. The death rate for both counties by this type of injury showed an increase over the study period.

In Miami-Dade County, stabbing-related deaths were proportionally equal among Black Non-Hispanic (42%) and Hispanic (42%) victims. In Broward County, White Non-Hispanics (57%) were more likely to be victims of stabbing related homicide than Black Non-Hispanics (29%) and Hispanics (14%).

Homicide by gunshot far surpasses other types of homicide injuries for victims older than 14 years of age, especially among young individuals with aged between 14 and17 and 18 to 24 years old with 81% and 92% respectively.

In Broward County gunshot-related homicide is still the most common at 64% but stabbing (12%) and other types of homicidal injury (15%) were higher than in Miami-Dade County. Younger groups follow a similar pattern in Miami-Dade County; the vast majority of victims are gunshot-related. In Broward County, proportionally more (88%) adolescents between 14 and 17 years of age died from gunshot-related injuries than in Miami-Dade County. Twenty six percent of homicide victims younger than 14 years of age died from gunshot-related injuries.

**Socioeconomic Factors**

There was a significant inverse relationship between homicide rates and the proportion of adult individuals, 25 years and older, with high school or higher education, (r=0.67, p < 0.01). Based on our analysis, the more educated the state population, the lower the risk of dying by homicide. Poverty (r = 0.56, p < 0.01) and unemployment rates (r = 0.54, p < 0.01) are both significantly associated with homicide. The homicide rates are consistently higher in those states with more population living under the poverty level and more unemployed individuals.

## Discussion

The probability of being a victim of homicide in Miami-Dade County is approximately two times higher than in Broward County and from 1.6 to 2 times higher than the entire state of Florida. Between 2004 and 2007 the risk of dying in the area was more than two times higher than in the rest of the nation. There has been a gradual increase in the incidence of homicide in both Miami-Dade County and Broward County since 2004. Florida’s murder rate increased 27% in the first half of 2006 with homicides in some counties running at record-high levels; homicide rates increased 81% in Palm Beach County, 71% in Duval, and 42% in Miami-Dade County based on Crime Control Digest report.^4^

In 2006 the FBI reported that metropolitan areas in the U.S. had a homicide rate of 6.2 deaths per 100,000 people, two times higher than rates for nonmetropolitan counties (3.1).^5^ In the same year, the homicide rate for Miami-Dade County (11.45 deaths per 100,000 population) was two times higher than the average for all metropolitan areas in the nation. According to the Florida Department of Law Enforcement’s 2006 Uniform Crime Report, Miami-Dade County had the second highest murder rate in Florida among those counties with more than one million population.^6^

Black Non-Hispanics were killed by gunshot proportionally more than any other race/ethnic group in both counties. Black Non-Hispanic victims in Miami-Dade County were considerably younger than White Non-Hispanics and Hispanics. In Broward County, the Black Non-Hispanic victims’ average age was not significantly different than that in Miami-Dade County, but was younger than that of White Non-Hispanics in Broward. According to the Florida Department of Health, in 2006, Blacks were murdered at a significantly higher rate, than Whites in both Miami-Dade County and Broward County.^7^

A substantial portion of the homicide victims in both Broward County and Miami-Dade County were adolescents. In fact, there has been an increased risk of dying by homicide in young Miami-Dade County Black Non-Hispanic and Hispanic residents over the period of this study. Nationally, homicide is the second leading cause of death for Blacks aged between 10 and 14 years of age and the first for Blacks aged between 15 and 19 years of age. For Hispanics aged between 10 and 14 years old, homicide is the third leading cause of death and the second leading cause for adolescents aged between 15 and 19 years old.^8^

The increased number of victims among Black Non-Hispanics identified in our study is consistent with published national statistics. According to the U.S. Department of Justice, Black males between 18-24 years of age had the highest homicide victimization rates; the rate doubled for black males aged 25 and older.^9^ When considering the causes of death in individuals between 25 and 44 years of age the death rates from homicide in black males are 3 times that of Hispanics. Homicide has been reported to be the leading cause of death in African American males ages 18-64.^10^

Previous research has identified gunshot wounds as a primary cause of death in young victims,^11,12^ with higher numbers of firearm-related deaths in states with less-stringent gun laws and high rates of gun ownership, like Florida.^13,14^

Victims of gun-related homicides, represent the vast majority in both selected counties. In fact, Miami-Dade County and Broward County have a higher prevalence of this type of injury than the entire nation, according to the Bureau of Justice 2007.^9^

In both Miami-Dade County and Broward County approximately 60% of all gunshot victims were Black Non-Hispanics. A study conducted in Newark, New Jersey, found that 87% of gang related victims of homicide were African American and 12% were Hispanics. Nationally, juvenile homicide victims were overrepresented in gang-related killings.^11^

In January of 2007, Demarzo of the Miami Herald filed a report on the rise of young black male homicide victims. She described the shooting death of an 18 year old in Broward County to mark the end of a “bloody 2006”. In Miami-Dade County, almost half of the homicide victims in 2006 were young black males according to the county’s Medical Examiner.^15^

“Homicides are most often committed with guns, especially handguns. In 2005, 55% of all homicides were committed with handguns, and 16% with other guns.^16^Guns are the weapon of choice nationwide and Miami-Dade County is no exception. In fact, according to Miami-Dade County Police Department’s Director Robert Parker, “unfortunately the weapon of choice is a high-powered firearms in this community”, as quoted by Hargot from the Miami Sun Post.^17^

Miami-Dade County police confirmed that the recent homicides have been characterized by drive-by shootings and use of assault rifles.^18^ The gun violence has not escaped the attention of Miami-Dade police agencies or local communities,^19^ but programs such as gun buybacks and arresting juveniles for minor offences have failed to decrease firearm-related violence.^20^ Firearm injury is a life threatening event with a high lethality, and it should be the target of primary prevention.^21^

Economic difficulties or poverty and many other associated issues have long been connected with substance abuse. Furthermore, according to the most recent Arrestee Drug Abuse Monitoring (ADAM II) program data there is also a significant connection between substance abuse and violent crime nationwide.

Every year over 10,000 individuals are the victims of gun-related homicide in the U.S. Though these guns are often illegally obtained by criminals the majority were, at some point, purchased or manufactured legally in states such as Pennsylvania, North Carolina Georgia and Florida were the gun laws are not strong.

Previous research shows that there is a direct correlation between overall violent crime, the rate of homicide, and level of city disadvantage particularly cities in which poverty exists in clustered areas.^22^

Education, poverty level, and unemployment, appear to be relevant factors associated with homicide as reported here. States with higher poverty rates and lower educational levels are consistently those showing higher risk of dying by homicide. Miami-Dade County has an overall rate of poverty above the state and national average, the county also has a significant disparity in poverty levels when broken down by race. While the national poverty rate is 13.4%, Miami-Dade County in 2007 had a poverty rate of 16.4% which is broken down as follows: 10.2% for non-Hispanic Whites, 15.7% for Hispanics, and 25.6% for Black non-Hispanics.^23^

This study clearly shows a significant increase in the number of deaths by homicide in Miami-Dade County and Broward County in recent years. In particular, we report that the risk of dying by gunshot in young Black Non-Hispanic and Hispanic residents is significant. The devastation homicide inflicts on black teens and adults is a national crisis, yet it is all too often ignored outside of affected communities.^24^ Any intervention aimed at decreasing violence must pay attention to the social justice’s paradigm in place.

Recently, members of law enforcement and the judiciary have become concerned about a loophole in state law that allows convicted felons to keep a concealed weapons permit, even if convicted of using a deadly weapon during a crime. Miami-Dade County judges and prosecutors have vowed to lobby state legislators to close this loophole as one way to decrease firearm-related violence.^25^The status quo must be changed; comprehensive and coordinated approaches including stricter legislation of gun control is demanded.

Dissemination of these facts about homicide and its impact in our communities should be a priority not only for the law enforcement agencies and legislators, but also for educators and health professionals. It is imperative to increase awareness of this social problem affecting all layers of society; education is crucial.

Though we hope the results of this study will serve to raise the level of awareness regarding the rising incidence of homicide in South Florida it does suffer from some limitations. In particular, the classification of the victims by race/ethnicity in the medical examiners’ database was initially limited exclusively to “White” and “Black”. Victims were not originally classified as Hispanic in counties where this is a predominant ethnic group. Reclassification of victims as Hispanics in our study might not be error-free, therefore underrepresentation could have occurred. In addition, information regarding the type of injury and zip codes in the original database is incomplete.
